# Filaggrin and atopic march

**DOI:** 10.11613/BM.2019.020501

**Published:** 2019-06-15

**Authors:** Ivana Čepelak, Slavica Dodig, Ivan Pavić

**Affiliations:** 1 Department of Medical Biochemistry and Hematology, Faculty of Pharmacy and Biochemistry, University of Zagreb, Zagreb, Croatia; 2 Department of Pulmonology, Allergology and Immunology, Children’s Hospital Zagreb, Zagreb; School of Medicine University of Zagreb, Croatia

**Keywords:** atopic dermatitis, atopic diseases, filaggrin

## Abstract

There is an increasing number of experimental, genetic and clinical evidence of atopic dermatitis expression as a pre-condition for later development of other atopic diseases such as asthma, food allergy and allergic rhinitis. Atopic dermatitis is a heterogeneous, recurrent childhood disease, also present in the adult age. It is increasingly attributed to systemic features and is characterized by immunological and skin barrier integrity and function dysregulation. To maintain the protective function of the skin barrier, in particular the maintenance of pH, hydration and antimicrobial functions, the filaggrin, among others, plays a significant role. Filaggrin is a multifunctional, histidine-rich, insoluble protein. The lack of filaggrin is associated with various cutaneous (*e.g. ichthyosis vulgaris*, allergic contact dermatitis) and non-cutaneous (*e.g.* diabetes, inflammatory conditions of the gastrointestinal tract) diseases and may be a result of genetic, immunological factors combined with environmental factors. In this review we summarised (emphasized) recent findings in understanding the role of filaggrin in atopic dermatitis and other diseases, participants in the atopic march.

## Introduction

**Figure 1:**
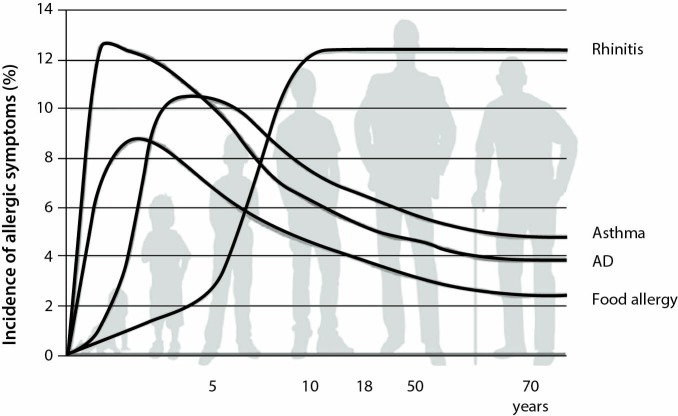
Atopic march. AD – atopic dermatitis. Modified according to (*6*).

The term atopy, which was first coined by Coca and Cooke in 1923, represents immunoglobulin (Ig) E-mediated type I hypersensitivity reactions (*1*). Childhood atopic diseases typically develop in mucosal surfaces, such as skin, respiratory and gastrointestinal system, showing a high degree of comorbidity. Prevalence of atopic diseases, including atopic dermatitis (AD), asthma, allergic rhinitis (AR), and food allergies (FA), has increased in recent decades and currently affects up to 20% of the population worldwide (*2*). These diseases seem to be closely related. The process by which several atopic diseases are interconnected throughout life, in this case “progression” of AD into asthma and AR is an epidemiological phenomenon commonly referred to as “atopic march” (*3*). The term refers to “time progression” from AD to asthma and AR during childhood, suggesting that AD is an “entry point” for subsequent atopic diseases (*4*-*6*) (Figure 1). However, there are opinions that AD is not a causal factor for the atopic march, and that the sequence of events does not always have to be the same (*7*). Belgrave *et al.* in addition to some other researchers, question the paradigm of the atopic march, considering it’s too simplified and needs to be revised (*8*, *9*). They explain it by the fact that most of the studies involving atopic march have been conducted based on cross-sectional statistical analysis on the general population, not taking into account the heterogeneity of the chronology of the development of symptoms. They hypothesized that children with AD that later develop asthma and AR may represent a specific phenotype. Although mentioned in the context of the atopic march, evidence of AD related to FA is unclear and deficient (*10**)*). According to Hill *et al*. atopic diseases have some common multiple genetic and environmental predisposing factors, share immune characteristics of one or more allergen-specific Th2 responses, and include type 2 effectors phases in which specific IgE, granulocyte activation and other inherited factors occur (*11*). Most of the investigations of the causal nature of “progression” are aimed at seeking evidence that supports the premise that AD of early childhood encourages the development of FA and respiratory allergies *via* systemic sensitization resulting from impaired skin barrier function. Thus, the hypothesis that the main cause of atopic diseases is a defect in epithelial barrier integrity is accepted (*12*, *13*). The most investigated causes of the epidermal skin barrier impairment in AD, as the initiator of atopic march, are a lack of filaggrin (FLG) associated with decrease of ceramide and significant activation of epidermal proteases (*14*).

In this review, we focus on the FLG molecule and its importance in maintaining normal skin barrier function and on current knowledge of the significance of FLG deficiency in diseases associated with atopic march. An overview of the recent knowledge of the nature of these conditions and their association with the role of FLG will be given. Appropriate scientific papers were selected on the PubMed using the following headings and keywords, and their combinations: filaggrin, profilaggrin, filaggrin mutations, atopic dermatitis, eczema, epidermal dysfunction, allergic rhinitis, food allergies and asthma. The search included epidemiological, genetic and clinical examinations, mainly on patients of various ages, regardless of the ethnic group.

## Atopic march development

Causes of skin barrier dysfunction can be intrinsic and extrinsic. Of the intrinsic causes, apart from the mutation of the gene encoding FLG, mutations of numerous other genes, such as the *SPINK5* gene encoding a serum protein (Kazal type 5), and *CDSN* genes encoding corneodesmosin were investigated (*15*, *16*). Extrinsic causes are scratching, environmental irritation, microbes, viruses, protein allergens, *etc*. The reactions that follow skin barrier impairment and precede Th2 inflammation and IgE synthesis include various cytokines (*e.g.* interleukine (IL)-25, IL-33 IL-4, IL-13, IL-5, *etc*.) and various cell types (*e.g*. basophilic and eosinophilic granulocytes, dendritic cells, mast cells and other types of cells). However, the TSLP (thymic stromal lymphopoietin), a type I cytokine produced by keratinocytes through the protease-activated receptor*-*2 (PAR-2) mediated nuclear factor *kappa* B (PAR-2/NF-kB) pathway, has the greatest significance. This cytokine has an essential role in the initiation of allergic inflammation in the skin. It activates Langerhans cells that promote differentiation of naive T-cells in Th2 cells in lymph nodes (*16*, *17*). In addition, TSLP reduces the FLG expression in human skin, and acts as a sensory neuronal activator, resulting in itching (*18*, *19*). Some authors consider that TSLP in the circulation can be a marker of lung responses to allergens. The TSLP could also be an important therapeutic target for reducing asthma and AR in children with AD (*20*).

**Figure 2:**
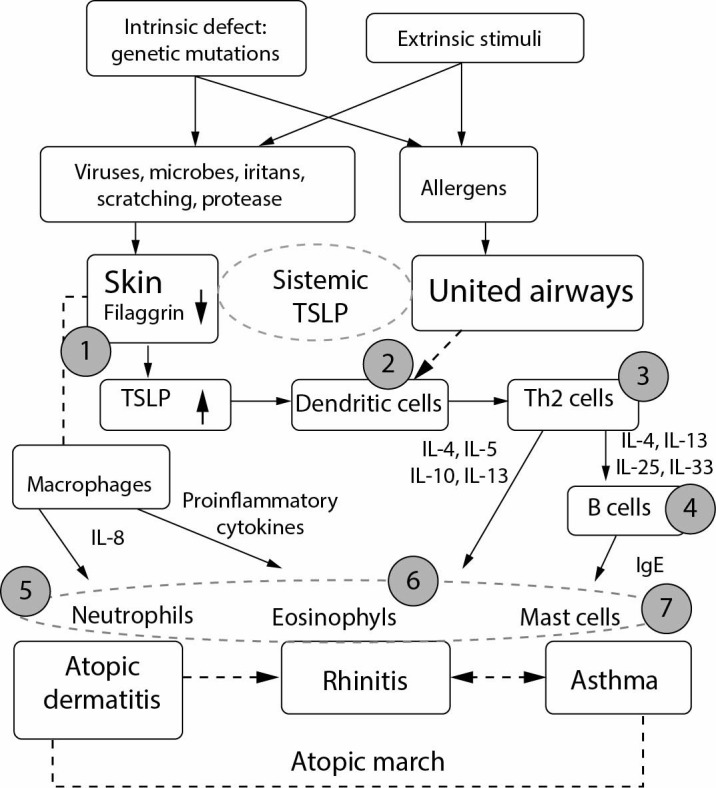
The mechanisms of atopic march. Inherited or acquired filaggrin deficiency results in immune and inflammatory changes. In addition, inflammatory and immune changes may result in filaggrin deficiency. Therefore, there is a positive feedback loop. Skin barrier impairment caused by deficiency of filaggrin; reduced filaggrin function leads to increased activity of TSLP, which through dendritic cells acts to promote both Th2 cell adaptive immune responses and Th2 innate immune cell response (marked as 1). Allergen uptake and presentation to dendritic cells in a Th2 context (marked as 2). Th2 cell expansion and activation (marked as 3). B cells activation and IgE switch (marked as 4). Neutrophil recruitment and activation (marked as 5). Eosinophil recruitment and mediators’ release (marked as 6). Immunoglobulin E binding to the high-affinity IgE receptor (FcεRI), on mast cell and basophil receptors (marked as 7). Th – T helper. IL – interleukine. IgE – immunoglobulin E. TSLP - thymic stromal lymphopoietin.

Genetics play a greater role predisposing AD to AR and AD to asthma than environmental factors (*21*). In addition, the relationship between AD and asthma, and between asthma and AR, does not depend on common environmental factors of early life. Therefore, various genes were studied such as genes responsible for epidermal integrity (FLG, CLD, *etc*.), genes responsible for the functioning of the immune system (*e.g*. IL-13, IL-33, *etc*.), the non-specific immune response participant genes (*e.g*. glutathione-S-transferase) and the genes responsible for chronic tissue inflammation (*e.g*., *COL29A1* gene encoding collagen, *ADAM33* gene expressed in fibroblasts and smooth muscle cells, *etc*.) (*5*, *10*). Despite differing opinions on the paradigm of atopic march, it is currently accepted that the primary disorder is the disturbed structure of the *stratum corneum* (SC) or barrier function. Thereafter, an increased absorption of allergens (allergic sensitization) predisposes the patients to the development of other atopic diseases. Genetically or acquired loss of FLG with epidermal structure impairment results in significant changes in both skin hydration and increased skin pH. Consequently, serine proteases activity is increased. Serine proteases *via* suitable mediators recruit and activate innate cell types that release IL-25, IL-33 cytokines, and particularly cytokine TSLP. The TSLP promotes the activation of dendritic cells, which migrate into the lymph organs and activate naive T cells and B cells, finally resulting in Th2 immune response. Increased penetration of allergens due to the disturbed structure of the skin barrier contributes to the Th2 immune response. The TSLP and the pro-inflammatory cytokines migrate to various pathways entering into systemic circulation and consequently in the airway and nasal mucosa resulting in asthma and AR. On the other hand, Th2-cytokines such as IL-4, IL-13, IL-25, as well as cytokines of other T-cell subtypes, whose number increases in AD depending on the subtype of disease, may suppress FLG expression in keratinocytes or aggravate inborn barriers defect through positive loop feedback. The pattern of atopic march development, including FLG deficiency and currently known major factors, is illustrated in Figure 2.

## What is filaggrin?

Filaggrin (from “FILament AGgregating pRoteIN) is a structural, S100 calcium-binding epidermal SC protein (*22*). It binds the intermediate filament of keratin producing micro-fibrils and is responsible for the normal SC function. It is a product of the proteolytic activity of the serine protease group enzymes to a large (molecular mass (Mm) > 400 kDa), insoluble and functionally inactive precursor molecule - profilaggrin. Profilaggrin is a complex, highly phosphorylated molecule rich in histidine and is the main constituent of the F-type keratohyaline granules of the *stratum granulosum* (SG). The profilaggrin expression is under the control of transcription factors such as the Activator protein 1 (AP-1) transcription factor family members (*23*). Profilaggrin encodes the *FLG* gene, one of the genes that encode proteins with epidermis formation function. It is localized on the short arm of chromosome 1 and belongs to the group of so-called epidermal differentiation complex (EDC), located in region 1q21. During the epidermal differentiation process of keratinocytes in the boundary between SG and SC, the profilaggrin molecule is dephosphorylated under the influence of phosphatase and becomes more soluble (*24*). Further action of different proteases results in cleavage of the profilaggrin molecule in several stages, separating the N- and C-terminal domains of the molecule, splitting the central part into trimers and dimers, and finally into 10-12 functional monomers - the FLG molecules. Each FLG molecule consists of 324 amino acids, with an Mm of 37 kDa. The resulting FLG monomers aggregate keratinic filaments along with the catalytic activity of the transglutaminase-1 enzyme, resulting in the marked morphological and cytostructural changes of keratinocytes (become flattened corneocytes). In addition to FLG, many other proteins, such as loricrin, involucrin, small proline-rich proteins, are involved in the construction of the cornified envelope (*25*). In the further course of epidermal differentiation, separation of the part of FLG from the structure of the cornified envelope occurs resulting in posttranslational conversion of arginine residues into the citrulline residues. The transformed FLG molecule is subject to the action of caspase-14 and is degraded. The FLG degradation products include a mixture of hygroscopic amino acids such as glutamine, histidine, alanine and their derivatives such as trans-urocanic acid (tUCA), degradable product of histidine and pyrrolidone carboxylic acid (PCA), degradable glutamine and glutamic acid product (*26*). These compounds along with some other such as hyaluronic acid, lactate, sodium, potassium, magnesium, phosphate, calcium, and citrate are the main constituents of so-called natural moisturizing factor (NMF) of the skin (*27*). The main characteristics of profilaggrin, FLG and its degradation products are presented in Table 1.

**Table 1:** Main characteristics of profilaggrin, filaggrin, and its degradation products (amino acids)

**Profilaggrin** **(Mm > 400 kDa)**	**Filaggrin** **(Mm ≈ 37 kDa)**	**Amino acids** **(NMF)**
Main constituent of keratohyalin granules, calcium-binding polyprotein, enucleation during cornification, keratinocyte-calcium signalling	Contribution to SC structure and function, aggregation of intermediate filaments, inhibition of transepidermal water loss	Epidermal hydration, acidification, photo protection, immunomodulation, antistaphyloccoccal effect
SC - *stratum corneum*. NMF - natural moisturizing factor. Mm – molecular mass. According to reference (*27*).		

To date, approximately 60 loss-of-function FLG mutations have been identified, with a difference in the spectra of mutations between different populations (*28*). The frequency of FLG mutation in the general population is 8-10%. The most common mutations in the European population are the R501X and 2282del4 mutations, resulting in premature transcription or inability to appropriately process profilaggrin in FLG. Mutations R244x and S3247X were found with significantly lower frequency (*29*). Lack of functional FLG due to the FLG gene mutation and to inflammatory and proinflammatory mediators that may affect FLG expression (acquired deficiency), results in a dysfunction of processes necessary for the corresponding protective SC role.

## Filaggrin and skin barrier

The skin barrier function is largely dependent on SC. The interior of the corneocytes consists mainly of keratin filaments aggregated by FLG, which is one of components that provide a scaffold for the extracellular lipid matrix. Filaggrin degradation products maintain in part for the both, water-holding capacity and acidic pH of the SC (*25*). Natural moisturizing factor components are essential for skin barrier integrity, *i.e*. for skin hydration, skin pH modulation, immunosuppressive properties, antimicrobial defence, photosensitivity and skin elasticity (*27*). Because of water loss, there is the loss of skin elasticity. Inadequate humidity of the skin can also stimulate the production of proinflammatory mediators. The skin pH, normally ranging from 4.5 to 5.5, is increased. Endogenous causes of skin pH increase are amino acid deficiency, disruption of decomposition of amino acids into tUCA and PCA, degradation of fatty acid from epidermal phospholipids, and changes of Na^+^/H^+^ transmembrane transport. Increased pH results in increased activity of serine proteases that can activate some cytokines and cause inflammatory reactions. The exogenous causes of pH increase include the microbial skin flora metabolites, the activity of sweat glands, and sebum derived compounds. Changing pH from neutral to alkaline values favours the development of various pathogens on the skin surface, most commonly *Staphylococcus aureus* and *Candida albicans*. All these changes result in reduced SC cohesion, enabling penetration of allergens and other pathogens in the skin.

Filaggrin mutations, *i.e*. FLG deficiency is also associated with various skin diseases (*ichthyosis vulgaris*, eczema herpeticum, atopic dermatitis, periodic infection with *Staphylococcus aureus*, allergy to nickel, allergic contact dermatitis in combination with AD, eczema, *etc*.) but also non-skin diseases (*e.g*. asthma in combination with AD, peanut allergies, allergic rhinitis, diabetes) (*30*).

## Therapeutic approaches

New therapeutic approaches directed to epidermal barrier defects are being attempted (*31*). Primary intervention in the treatment of diseases involving FLG deficiency is directed to improving profilaggrin processing into FLG, the recombinant FLG supplementation, as well as the NMF supplementation. It is believed that such a therapeutic approach could have a significant effect on a broader range of diseases associated with FLG gene mutation carriers, persons with FLG deficiency caused by inflammation, or in persons with genetic or inflammatory enzyme variants that allow the conversion of profilaggrin into FLG. Most of the studies of the effects of such a therapy have been made in both animal models, and *in vitro* tests; the results are promising. However, a beneficial effect on the development and progression of atopic march in human samples has yet to be established. For more reliable conclusions about the effectiveness of such therapy, longer periods of disease monitoring are required.

## Atopic dermatitis and filaggrin

**Figure 3:**
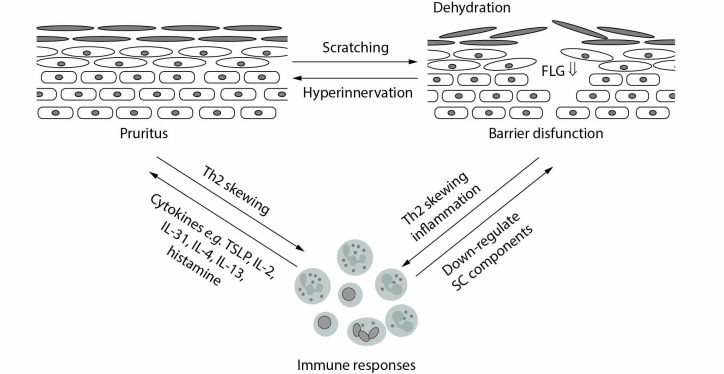
The relationship between itching, skin barrier abnormalities and immune dysregulation. Scratching due to itching may aggravate skin damage. Dry skin stimulates itching by increasing the density of epidermal nerve fibres. Scratching also promotes Th2 chemokines, eosinophil recruiting chemokines, and TSLP. Obviously, the immune response can induce itching through the secretion of numerous cytokines that can act as a pruritogens. FLG – filaggrin. TSLP – thymic stromal lymphopoietin. Th – T helper. IL – interleukine. SC – *stratum corneum*. Modified according to (*35*).

Atopic dermatitis is a common multifactorial skin disease of childhood, and it is postulated as the first manifestation of the atopic march (*32*). The prevalence of AD has increased in recent decades and currently affects up to 20-30% of children and to 3% of adults worldwide (*3*). It has some common features with FA, asthma and AR including abnormality of skin barrier function, allergen sensitization and type 2 immune response (*33*). Clinical manifestations of AD are the result of synergistic action of genetic and immune mechanisms in combination with environmental factors (climate, nutrition, obesity, *etc*.). The disease is characterized by inflammation, immune dysregulation, skin barrier dysfunction, and in most but not all (10-40% of AD patients) with IgE-mediated sensitization to food and environmental allergens. Major symptoms include xerosis, eczema lesions, dry itchy skin, and chronic or relapsing dermatitis. Consequently, the continuous itchy-scratch cycle results in trans-epidermal water loss and secondary skin infections (*34*) (Figure 3).

The form of AD with a normal serum IgE concentration is known as a non-atopic or intrinsic AD. It is not related to other atopic diseases, but the clinical picture does not differ from atopic or extrinsic AD (*36*). In addition to skin, systemic sensitization is also associated with AD. According to Brunner *et al*. various comorbidities are investigated, such as association with cardiovascular, neuropsychiatric, malignant diseases, but proof of this connection requires further longitudinal studies (*37*). Traditionally, the dysfunction of the adaptive immune system is considered a primary pathogenic mechanism that initiates AD. Specifically including Th2/Th1 imbalance and inflammation, which secondary results in skin-structure barrier impairment, which is considered an epiphenomenon. In a number of studies that followed the discovery of FLG gene mutation, an epidermal barrier impairment including FLG deficiency was considered as a primary pathogenic initiation mechanism of AD (*38*).

According to Thyssen *et al*. the important observations that put FLG into the centre of pathogenesis of AD are: impaired FLG values in skin with or without lesions in patients with AD, the fact that approximately 50% of patients with moderate or severe AD have at least one FLG mutation, that a decrease in the number of copies of FLG increases the risk of AD, that the decreased values of FLG were demonstrated in skin lesions despite the absence of FLG mutations, and that some of the existing therapies can repair FLG values (*39*). The development of AD, along with genetic and immune mechanisms, is also strongly influenced by environmental factors such as industrialization, stress exposure, obesity, excessive use of antibiotics, sedentary lifestyle, *etc* (*40*).

Specifically, the damaged skin-barrier function allows penetration of various allergens/haptens, environmental pollutants, and toxins resulting from bacterial colonization on the one hand, and on the other hand by sensitization to allergens. For example, it has been shown that phthalate metabolites, common ingredients of cosmetic products, increase the risk of AD, and that children with FLG P478S mutations absorb phthalates (*41*). The pathogenesis of AD is also linked to *FLG-2* gene mutations that encode the filaggrin-2, a protein similar to FLG by localization, amino-acid composition and biochemical properties (*42*). Also, other genes may play a role in the pathogenesis of AD, *e.g*. *HRNR gene* encoding the hornerin, *RPTN* gene encoding the repetin, a *SPINK5* gene encoding Kazal-type 5 serine protease, LOR gene encoding loricrin, and other genes by location close to epidermal differentiation complex (*43*-*45*). In addition, gene mutations that are involved in the immune response (*e.g*. IL-4, IL-5, IL-13, IL-18, IL-31, IL-4RA, a binding protein gene *e.g*. claudin (CLDN1) and desmoglein 1 protein (DSG1) have been investigated (*46*, *47*). In the focus of the investigation of pathogenesis of AD and atopic march is also a study of various epigenetic modifications such as methylation of DNA, FLG, TSLP, and variants of enzymes involved in synthesis and profilaggrin processing (*48*). Most studies have shown that the lack of structural and functional epidermal molecules does not only disturb the structure of the barrier, but is also a mediator of immune and allergic processes. There is a reciprocal relationship between the skin barrier dysfunction and the immune response in pathogenesis of AD. Therefore, AD can still be considered as a disease of skin barrier impairment and immune disease (*49*). Mechanisms of AD progression into other atopic diseases are not quite clear. One of the participants in this process is the platelet activating factor (PAF), an endogenous phospholipid inflammatory mediator released by various types of cells, *e.g*. alveolar macrophages in allergic and inflammatory conditions (*50*). Release of PAF is associated with increased vascular permeability, eosinophilic granulocyte attraction, and bronchoconstriction.

## Filaggrin, asthma, and allergic rhinitis

Data on FLG expression in human digestive tract and unified respiratory system are contradictory and more often negative (*51*, *52*). Moreover, mechanisms linking FLG variants with asthma, AR or FA are not entirely understandable. However, based on some animal models, it is considered that skin sensitization, including the Th17 cell subpopulation, facilitated by acquired FLG defects or mutations can indirectly result in local but also systemic inflammation in distant organs (*53*, *54*).

## Asthma

An estimated 300 million people worldwide suffer from asthma (*55*, *56*). Asthma is caused by genetic predisposition and various external stimuli (*e.g*. airborne allergens), which result in partial or full reversible bronchoconstriction. It is often presented differently in children and adults; there are different phenotypes such as intermittent, persistent, aspirin sensitive and severe form. Symptoms mainly involve severe breathing, chest tightness, dyspnoea and cough, especially at night and in the morning (*55*). Asthma is the result of a disturbed interaction of the respiratory epithelium, the innate and adaptive immune system and the environmental factors. In this regard, a large number of genes, particularly genes responsible for the Th2 cytokine expression in Th2 cells (IL-4, IL-13, IL-5), other T-cell subtypes, dendritic cells, eosinophil and neutrophil granulocytes, mast cells, macrophages and IgE were investigated (*57*). In the pathogenesis of asthma, the role of ADAM33 protein, responsible for bronchial hyperresponsiveness, stimulation of smooth muscles of the airways, fibroblast proliferation and cytokine production has been studied (*58*). Moreover, the nitric oxide vasodilator system, various growth factors (*e.g*. transforming growth factor-beta, granulocyte-macrophage colony-stimulating factor), inflammatory lipid mediators such as leukotrienes and prostaglandin D2, TSLP which induces key changes in dendritic cells and other molecules were also investigated (*59*). Palmer and colleagues first described the relationship between the R501X and 2282del4 variants of FLG genes and asthma in persons with AD (*60*). Case control, family and population studies and/or other FLG mutations, which then followed, more clearly indicate the meaning of a defective epidermal barrier in pathogenesis, primarily AD, and then other atopic disorders – AR and asthma (*61*, *62*). Generally, the results of investigating the association of these conditions with groups of subjects of different geographic origin show that those patients with FLG mutation have a moderately higher risk of developing asthma than those without genetic mutation, regardless of AD status (*63*). The risk is significantly higher (3 to 6-fold) in patients with AD and in carriers of FLG mutation (*64*). It appears that the presence of a particular FLG mutation also has implications for some clinical features of asthma such as the severity of the disease, the number of acute exacerbations, the degree of airway obstruction, and the quantity of asthma control medications to control the disease (*65*).

## Allergic rhinitis

Rhinitis is one of the most common chronic conditions in children, but also in adults. It is an inflammation of nasal mucosa, characterized by nasal congestion (rhinorrhoea), itching, sneezing, and nasal blockage. Causes of rhinitis may be allergies, infections, drugs, hormones, various irritants, and may also be of idiopathic origin (*66*). Allergic rhinitis is characterized by a Th2 immune response including mediators such as IL-4, IL-5, IL-13, whose activity results in the formation of IgE and recruitment of eosinophilic and basophilic granulocytes, mast cells, which further release different mediators, such as histamine and cysteinyl leukotriene, as a vasoactive substance (*67*). About 40% of AR patients had asthma, and up to 80% of asthmatic patients reported AR (*68*). These data are not surprising since, besides the anatomical connection, the inhaled air is heated, moistened, and filtered in the nose to maintain homeostasis of the respiratory system (*69*). In the literature, therefore, more work is devoted to the connection of AR and asthma. Of the mechanisms considered in this connection, it seems that the most acceptable one is the systemic inflammatory response, as cited by Chawes (*70*). The same author found a significant association of FLG mutations with AR, but not with non-allergic rhinitis. Other authors also report about such association (*71*, *72*), which supports the hypothesis of the primary role of epidermal barrier impairment in this disease.

## Open questions

Most of the scientific articles included in this review consider FLG deficiency, in particular caused by the FLG mutation, as one of the causes, the key risk factor, the AD modifier, and consequently the possible causes of atopic march.

The disruption of the skin barrier function associated with the lack of FLG in AD is, along with other known pathogenic factors, the basis for the allergic sensitization to food allergens and aeroallergens, and the consequent development of asthma and AR. The key questions in this context are 1) how to predict AD and when to prevent the occurrence of AD; 2) whether timely repair of skin barrier could prevent progression of AD and thus the possible development of asthma and AR; and 3) what an appropriate therapeutic strategy should include? To get the correct answers to these questions, it is necessary to define the best biochemical, preferably non-invasive markers for the detection and monitoring of the disease.

The ideal marker would be the one that would make it easier to set up a diagnosis, help assess the severity of the disease, identify the phenotypes AD, and predict the individual response to the therapy. However, the ideal marker does not exist.

Moreover, laboratory assays currently used in practice are mainly focus on monitoring immune or inflammatory changes (*e.g*. total and specific IgE, eosinophilic granulocyte counts, cytokine release assays) and less to control of skin-barrier function impairment including FLG deficiency. These are for example the determination of TWEL and pH of the skin (*73*, *74*).

For the last few decades, scientists have been trying to establish methods for detection of FLG deficiency, its degradation products, or products of profilaggrin processing as well as other barrier proteins. The analytical samples include skin biopsy, animal skin biopsy samples, human skin equivalents and reconstructed skin samples, samples collected by tape stripping technique and serum (*75*-*80*). Non-invasive *in vivo* methods for investigation of skin components are implemented in clinical practice. Among novel methods, Raman spectroscopy, an optical biopsy method, offers the possibility for real-time characterization of skin components, including FLG (*81*). The concentration of the NMF ingredients, for example amino acids and their derivatives is determined using the HPLC method (*76*). Activities of enzymes involved in the processing of profilaggrin into FLG, and FLG paraffin-embeded samples are also determined, by the use of immunohistochemical staining (*82*, *83*). In the studies there are also particularly important methods of genotyping of FLG, as well as proteomic analysis of FLG deficiency.

Of the non-invasive markers, it appears that significant potential for the clinical course of AD and therapeutic effect has TSLP expression determination using tape-stripping approach to sampling and mass spectrometry as well as determination of serum TSLP concentration by enzyme-linked immunosorbent assay (ELISA) (*84*, *85*). Also, thymus and activation-regulated chemokine (TARC), high-affinity ligand for CC-chemokine receptor 4 (CCR4) in serum or plasma of patients has been proposed as a marker of disease activity (*86*, *87*).

A significant number of biomarkers include direct or indirect evaluation of FLG deficiency. However, taking into account the multifactorial nature of AD, it is necessary to define more clearly the most reliable biomarker for a single phenotype AD (*12*). Accordingly, it will also be possible to apply appropriate therapy with the aim of repairing the skin barrier.

In addition to the appropriate preventive measures, effective therapeutic approach to maintain adequate skin barrier function and inflammatory control are also needed. Within primary non-pharmacological prevention, some authors advise avoiding various types of allergens, while some promote tolerance. Early sensitization in the first year of life, especially to food allergens, *e.g.* peanuts and milk, increases the risk for asthma to the third year of life of a children even 7-fold (*88*). Other authors state that sensitization to food allergens can develop before food is consumed, and that it occurs through inflammation of the skin (allergens can be found in home dust and creams and oils used for the treatment of new-born babies) (*89*). It is therefore considered that early oral consumption of such food can lead to tolerance, and that transcutaneous exposure to food allergens can lead to food sensitization. Based on the described causal link between early sensitization through damaged skin barrier and later development of atopic diseases such as asthma and AR, Johanson and Hershey promote immune tolerance as a “therapeutic” strategy or early introduction of “allergic” food (*90*). It has been described that the permeability of new-born skin is increased due to the insufficient presence of ceramide, which is associated with increased risk of later development of AD (*91*). A review by Lowe *et al.* focused on skin as a goal for the prevention of atopic march includes the concept of preventive skin treatment with emollients from the first weeks of life to 6 or 8 months of age, *i.e*. prevention before the onset of symptoms of AD (*92*). Few well-designed larger trials are currently underway to demonstrate the long-term effects of moisturizing on the incidence of AD and on the incidence of FA and allergic airways disease. The authors discuss the advantages and disadvantages of such a strategy, as well as the choice of emollients, and their safety and acceptability (*92*).

As a part of the primary therapeutic prevention of further AD development, as well as prevention of progression in asthma and AR, methods for compensation of reduced FLG level or its degradation products (NMF) are also developed.

According to Irvine, the group of candidates for FLG replacement therapy would include patients with FLG mutations, patients with variation in FLG repeat numbers, intra-genetic copy number variation, patients with secondary reduced FLG (acquired deficiency), severe and persistent type of AD that have allergic sensitization and in the future could develop asthma, and patients with genodermatoses (*93*). Currently, about 80 clinical trials focused on topical AD treatment are ongoing, including drugs that would have the ultimate effect on FLG increase (*94*). Animal and human investigations of drugs focused on mutant alleles, drugs increasing FLG expression by promotion of a healthy allele, direct FLG substitution drugs, hence recombinant FLG are currently underway (*95*). Other therapeutic measures are the renewal of extracellular skin lipid profiles (various emollients with a mixture of ceramides, cholesterol, and fatty acids), regeneration of pH, and skin hydration. As immune dysregulation remains the key problem in AD and shares pathophysiological features with other atopic disorders, it is very important to control inflammation. Various biological drugs such as dupilumab (monoclonal antibody directed against the IL-4 receptor α subunit that blocks signaling of both IL-4 and IL-13), nemolizumab (target IL-13), ustekinumab and lebrikizumab (targeting IL-31) are in research (*31*, *96*, *97*). However, further investigations should be used to determine the timing of the initiation of preventive measures, the effectiveness of various forms of therapy, safety, and the mode of administration/dosage and the duration of the effect.

## Conclusion

It is indisputable that the interactions between impaired epidermal barrier and dysregulation of the innate and adaptive immune system, in association with environmental risk factors, are involved in pathogenesis of AD. Atopic dermatitis is the most commonly occurring disease in an early life, which is associated with increased risk of asthma and AR. The scientific literature is dominated by works that AD of hereditary or other origin are considered as an initiating event in the atopic march and the lack of FLG as a key component of the epidermal differentiation complex, as one of the first participants in pathogenesis of AD. In this context, if the skin barrier function is impaired, or if it is not repaired in the case of a pathogenic process already exists, it will be possible to provide a continuous allergen entrance, and the immune system will continuously produce antibodies. It is therefore expected that, along with various preventive measures recommended for maintaining the barrier function, therapeutic-oriented barrier restoration in this context of FLG replacement and AD prevention will minimize the risk of allergic immunization as well as the development of asthma and AR. To monitor the effect of such therapy and the therapy that is in use, it is also necessary to find suitable laboratory markers. The complete pathogenesis of AD and the pathway to explain atopic march, despite numerous new findings, including the significance of FLG deficiency, are still not quite clear due to their complexity and are in the focus of numerous researchers.

Accordingly, researchers should continue to work on the identification of molecular basis and environmental triggers of AD, on the association of the immune system with FLG deficiency, but also with other proteins of the epidermal differentiation complex. That will help to establish appropriate prevention and therapeutic algorithms to prevent or at least slow the atopic march.
